# Supplementary material for: Clinician-created educational video for shared decision-making in the outpatient management of acne
Source: PLoS One. 2022 Jul 8;17(7):e0271100. doi: 10.1371/journal.pone.0271100 (PMC9269380; doi:10.1371/journal.pone.0271100)
Supplement: S1 File — (DOCX) [file pone.0271100.s001.docx]

| 附表十 患者服用**Isotretinoin口服製劑**同意書 |
| --- |

病歷號碼：

茲證明本人 年齡 出生日期 年 月 日

身分證號碼:

地 址:

確認經由 醫院 醫師詳細告知服用

isotretinoin口服製劑 之主要可能副作用和處置方式。

本人完全瞭解isotretinoin口服製劑可能對本人的其他療法無效的嚴重性囊腫型及結節狀痤瘡有所幫助，但服用時必須要小心，特別要注意下列事項：

1. 女性患者服藥期間及停藥後至少 4 個星期內，絕對不可懷孕，因為在上述期間發生懷孕，則很可能產生胎兒畸形。

2. 服藥期間及停藥至少四個星期內，請勿捐血以免受血者產生畸胎。

3. 因為對肝腎功能不全之病人為禁忌，請病人需依醫師指示定期做血液、肝功能、血脂肪等方面之檢查。

4. 未經醫師許可，絕對不可將isotretinoin口服製劑轉予他人使用。

本人確實瞭解採取下列方法為絕對需要:

1. 女性患者在服用isotretinoin口服製劑 前必須驗孕以確定尚未懷孕。

2. 女性患者在服用isotretinoin口服製劑 之前的四個星期，治療期間及停藥後至少 4 個星期內，必須使用有效之避孕方法。

3. 萬一在上述期間發生懷孕，本人保證立刻通知 醫師。

雖然本人充分瞭解假使在上述期間發生懷孕可能導致之後果，本人仍然願意接受isotretinoin口服製劑 治療，並且願意承受這種危險性及有關注意事項。

為慎重計，特立此同意書。

日期: 民國 年 月 日，地點:

立同意書人:

（未達 20 歲之未成年需經法定代理人之同意）
