# Supplementary material for: Clinician-created educational video for shared decision-making in the outpatient management of acne
Source: PLoS One. 2022 Jul 8;17(7):e0271100. doi: 10.1371/journal.pone.0271100 (PMC9269380; doi:10.1371/journal.pone.0271100)
Supplement: S2 File — (DOC) [file pone.0271100.s002.doc]

**青春痘**


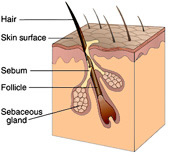
一、**病因**：

皮脂腺增大引起的皮脂分泌過剩，而這又是由於青春期皮膚隨著體內賀爾蒙而變化的 表現。皮脂腺的過度成熟肥大，造成極度的油性皮膚，此時倘若有任何惡因存在，容易使毛孔開口處的細菌繁殖。

二、**症狀**：

黑頭粉刺，白頭粉刺，膿皰，暗瘡，痘疤。

三、**治療**：

常見的治療青春痘外用添加物，大約可分為四類：1、油脂去脂2、剝皮劑3、抗生素製劑：賀爾蒙劑。

四、**注意事項**：

在皮膚醫學上，容易長青春痘的人，臉部常有油膩的感覺，可以使用肥皂(或香皂、要造、洗面皂等)，每天清洗臉部兩、三次，除去油膩的感覺。如果臉上黑頭過多，經常洗臉也可以使病況減輕。

五、**結語**：


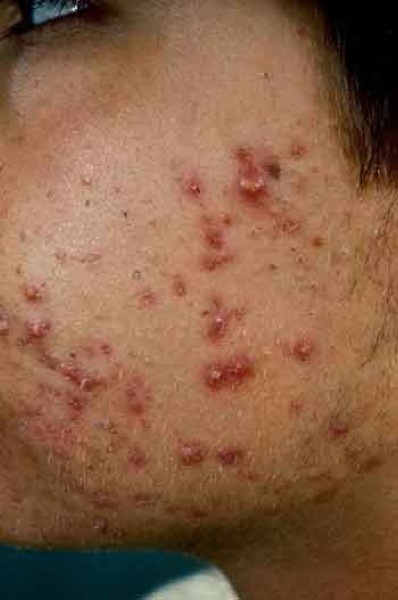
預防絕對勝於治療，其實只要明白青春痘的生成原因，預防起來也就沒那麼困難了。最重要的事做到幾點：保持臉部清潔，常化妝的女孩更不能偷懶。保持心情愉快，睡眠充足。不要隨便塗抹藥物，誤用不當的藥，可能使痘痘更多，甚至併發其他病變。注意日常生活飲食，少吃油炸類食物。總之，要想戰勝青春痘帶來的困擾，除了聽從醫師建議外，徹底做好臉部清潔工作室相當重要的，千萬別讓青春痘成為你青春年少的一大憾事！
