# Supplementary material for: Clinician-created educational video for shared decision-making in the outpatient management of acne
Source: PLoS One. 2022 Jul 8;17(7):e0271100. doi: 10.1371/journal.pone.0271100 (PMC9269380; doi:10.1371/journal.pone.0271100)
Supplement: S3 File — (DOCX) [file pone.0271100.s003.docx]

1. 口服A酸治療痘痘的主要機轉為何呢

A. 直接殺死痤瘡桿菌

**B. 造成皮脂腺萎縮，而減少出油**

2. 下列何者是口服A酸的禁忌症

A. 準備懷孕或已經懷孕

B. 哺餵母乳

C. 目前正在口服四環素類抗生素

D. 高血脂症

**E. 以上皆是**

3. 口服A酸身體會出現哪些副作用呢？

A. 嘴唇乾裂、皮膚乾燥

B. 眼睛乾燥、角膜炎

C. 肌肉、關節疼痛

D. 致畸胎性(尤其中樞神經系 統、心臟和大血管)

**E. 以上皆是**

4. 停止口服A酸後至少多久女性才可以準備懷孕呢？

A. 停藥後1日

B. 停藥後1星期(7天)

**C. 停藥後1個月**

D. 停藥後1年

5. 口服A酸需要檢查什麼項目呢？

A. 血球(包含白血球以及紅血球數量)

**B. 肝功能以及血脂肪**

C. 腎功能檢查

D. 肺功能檢查
