# Supplementary material for: Clinician-created educational video for shared decision-making in the outpatient management of acne
Source: PLoS One. 2022 Jul 8;17(7):e0271100. doi: 10.1371/journal.pone.0271100 (PMC9269380; doi:10.1371/journal.pone.0271100)
Supplement: S4 File — (DOCX) [file pone.0271100.s004.docx]

1. 相較於紙本的衛教，藉由影片所進行的衛教，你覺得如何呢？
2. 影片衛教比較清楚
3. 紙本衛教比較清楚
4. 影片和紙本衛教一樣清楚
5. 關於影片衛教你覺得可以加深對於內容的印象嗎
6. 非常同意
7. 同意
8. 沒差別
9. 不同意
10. 非常不同意
11. 關於影片衛教你覺得可以有效的瞭解藥物相關注意事項嗎？
12. 非常同意
13. 同意
14. 沒差別
15. 不同意
16. 非常不同意
17. 對於影片內容我的建議為~~(可以填寫個人的想法及建議)
