# Supplementary material for: Clinician-created educational video for shared decision-making in the outpatient management of acne
Source: PLoS One. 2022 Jul 8;17(7):e0271100. doi: 10.1371/journal.pone.0271100 (PMC9269380; doi:10.1371/journal.pone.0271100)
Supplement: Attachment — Submitted filename: Reviewer opinion and response.doc [file pone.0271100.s006.doc]

**Editor(s)' and Reviewer(s)' Comments to Author:**

**Editor**

Comments to the Author

1. Please ensure that your manuscript meets PLOS ONE's style requirements, including those for file naming.

**Response**:

Thanks for your kind reminder. We ensured that the manuscript meets PLOS ONE's style requirements, including those for file naming.

2. Please provide additional details regarding participant consent. In the ethics statement in the Methods and online submission information, please ensure that you have specified what type you obtained (for instance, written or verbal, and if verbal, how it was documented and witnessed). If your study included minors, state whether you obtained consent from parents or guardians. If the need for consent was waived by the ethics committee, please include this information.

**Response**:

Thanks for your kind reminder. The patients enrolled in our study filled out the quizzes, and questionnaires, and watched the educational video anonymously. The Institutional Review Board of Tri-Service General Hospital agreed that our study was exempt from patient consent (page 8, lines 16-19).

3. In your Data Availability statement, you have not specified where the minimal data set underlying the results described in your manuscript can be found. PLOS defines a study's minimal data set as the underlying data used to reach the conclusions drawn in the manuscript and any additional data required to replicate the reported study findings in their entirety. All PLOS journals require that the minimal data set be made fully available. For more information about our data policy, please see <http://journals.plos.org/plosone/s/data-availability>.

**Response**:

Thanks for your kind comment and reminder. We uploaded the data to the PLOS journal database.

4. We note that you have stated that you will provide repository information for your data at acceptance. Should your manuscript be accepted for publication, we will hold it until you provide the relevant accession numbers or DOIs necessary to access your data. If you wish to make changes to your Data Availability statement, please describe these changes in your cover letter and we will update your Data Availability statement to reflect the information you provide.

**Response**:

Thanks for your kind comment and reminder. We uploaded the data to the PLOS journal database.

5. Please note that supplementary tables (should remain/ be uploaded) as separate "supporting information" files.

**Response**:

Thanks for your kind comment and reminder. We followed the rule of PLOS ONE's requirements,

6. The topic is interesting (patient education, the communication of the risks and benefits of a therapy) but the work does not give many useful indications except that communication via video is more appreciated than a pamphlet with the same explanations.
(1). It may be interesting to evaluate the video shown in the link, but unfortunately the text is in Chinese

**Response**:

Thanks for your kind comment and reminder. We provided an educational video in English in the article (page 8, line 4).

(2). It would also be interesting to expand the discussion with some indications (if there are any) on the response of patients after watching the video, to know if their approach to therapy is different from those who do not undergo an educational video

**Response**:

Thanks for your kind comment and reminder. We revised and added patients’ responses in the article (page 12, lines 4-7).
 I think it would be easier to accept the manuscript at least by evaluating the video in English and and adding the reaction of patients to the educational video and some indications for doctors who want to adopt this approach.
In general, I agree with observations from reviewers.
Please provide clarifications.

**Reviewer**

Comments to the Author

Please use the space provided to explain your answers to the questions above. You may also include additional comments for the author, including concerns about dual publication, research ethics, or publication ethics. (Please upload your review as an attachment if it exceeds 20,000 characters)

Reviewer #1: -

1. Video is not in english and so not easy to evaluated correctly.

**Response**:

Thanks for your kind comment and reminder. We provided an educational video in English in the article (page 8, line 4).

2. Authors must clarify if dermatologists instructed in the same way the patients. Were they instructed before? This could be a bias of the study.

**Response**:

Thanks for your kind comment and reminder. The content consisted of the treatment guide for acne in Taiwan. Besides, the video was reviewed by another 5 dermatologists, and the content was revised 3 times. All doctors involved in SDM were instructed before the implementation.

3. Did authors make a power analysis fo choising the number of subjects to be tested?

**Response**:

Thanks for your kind comment and reminder. A two-tailed 99% confidence interval is ± 2.6 standard error (S.E.) wide. For a ±1 logit interval, this S.E. is ±1/2.6 logits, this gives a minimum sample in the range 4*(2.6)2 < N < 9*(2.6)2, i.e, 27 < N < 61, depending on targeting. Thus, a sample of 50 well-targeted examinees is conservative for obtaining useful.[1]

4. Video was editing following some standard guideline for patient education? If yes, please clarify in the text.

**Response**:

Thanks for your kind comment and reminder. The patient education video was edited followed the three steps as proposed by Ziade N. et al. in 2021 (page 8, line 1-2).[2]

5. Main limitations of the sutdies must be highlighted.

**Response**:

Thanks for your kind comment and reminder. We revised the limitations in our article (page 15, line 13-19; page 16, line 1-4).

**Reference**

1.	Khan MI. Recovery and stability of item parameter and model fit across varying sample sizes and test lengths in Rasch analysis with small sample. Social Science International. 2014;30(1):43.

2.	Ziade N, Arayssi T, Elzorkany B, Daher A, Karam GA, Jbara MA, et al. Development of an Educational Video for Self-Assessment of Patients with RA: Steps, Challenges, and Responses. Mediterr J Rheumatol. 2021;32(1):66-73. Epub 20210304. doi: 10.31138/mjr.32.1.66. PubMed PMID: 34386703; PubMed Central PMCID: PMCPMC8314883.
